# Double‐Duty Drugs: Repositioning Antipsychotics to Combat Bacterial Infections

**DOI:** 10.1002/cns.70724

**Published:** 2026-02-04

**Authors:** Navid Faraji, Mohammad Abavisani, Negar Ebadpour, Sercan Karav, Prashant Kesharwani, Amirhossein Sahebkar

**Affiliations:** ^1^ Mashhad University of Medical Sciences Mashhad Iran; ^2^ Immunology Research Center Mashhad University of Medical Sciences Mashhad Iran; ^3^ Department of Molecular Biology and Genetics Canakkale Onsekiz Mart University Canakkale Turkey; ^4^ Next‐Generation Translational Nanomedicine Laboratory, Department of Pharmaceutical Sciences Dr. Harisingh Gour Vishwavidyalaya (A Central University) Sagar Madhya Pradesh India; ^5^ Applied Biomedical Research Center, Basic Sciences Research Institute Mashhad University of Medical Sciences Mashhad Iran; ^6^ Centre for Research Impact and Outcome, Chitkara College of Pharmacy Chitkara University Rajpura Punjab India; ^7^ Biotechnology Research Center, Pharmaceutical Technology Institute Mashhad University of Medical Sciences Mashhad Iran

**Keywords:** antibacterial, antipsychotics, clinical studies, gut microbiota, *M. tuberculosis*, safety and efficacy

## Abstract

**Introduction:**

This article provides a comprehensive review of the antibacterial properties of antipsychotics, exploring proposed pathways and mechanisms of action. While experimental evidence supports certain mechanisms, such as efflux pump inhibition, others, including the impact on the respiratory chain in *M. tuberculosis* and cell wall inhibition, remain insufficiently substantiated.

**Methods:**

Current research primarily relies on in vitro experiments, with limited exploration of in vivo effects. The influence of antipsychotics on gut microbiota poses a significant concern, as alterations may lead to dysbiosis, which has been linked to various illnesses. Additionally, antimicrobial drugs can exert selective pressure, fostering resistance in bacterial strains.

**Results:**

Repositioning antipsychotics as antimicrobials is further complicated by the need for higher doses than those approved for therapeutic use in humans, raising safety concerns. The use of antipsychotics in non‐psychotic populations is particularly problematic due to a lack of proven efficacy and potential adverse effects, such as metabolic disturbances, movement disorders, and sleep issues.

**Conclusions:**

These challenges highlight the need for extensive in vivo and clinical studies to evaluate the antibacterial potential of antipsychotics, ensuring safety and efficacy. Careful monitoring and a balanced risk–benefit analysis are essential when considering antipsychotics for antimicrobial purposes.

## Graphical abstract

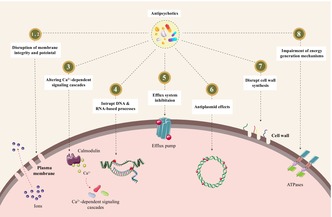

Antibiotic resistance has become a growing global issue in recent decades. Currently, there is an upsurge of multidrug‐resistant (MDR) organisms, which pose challenges in effectively treating infections. Addressing this issue necessitates the development of novel antimicrobial agents. Nonetheless, the process of discovering and marketing new drugs encounters numerous obstacles aside from being inherently costly; the process of introducing a novel drug and obtaining approval for its use among the general population would require a substantial amount of time. One of the shortcuts to overcoming these challenges is drug repositioning [1, 2]. The existence of several well‐known drugs, including hydroxychloroquine, sildenafil, tamoxifen, and thalidomide, illustrates that established medications can successfully address a variety of disorders beyond their initial intended uses [3, 4].

Antipsychotics have also gained attention in the field of drug repositioning. Phenothiazines, a first‐generation antipsychotic, have been shown to have an antimicrobial impact in various in vitro, in vivo, and in silico investigations, either by killing bacteria or by inhibiting their growth. The antibacterial activity of this category of antipsychotics is achieved through various mechanisms. These drugs can target the bacterial DNA and RNA, disrupting the DNA‐ or RNA‐associated processes (i.e., intercalation in the nucleic acid sequence), including cellular proliferation and plasmid replication. They can engage with the calcium‐binding protein (i.e., calmodulin) and hinder calcium‐related signaling pathways in living cells. Furthermore, phenothiazines possess amphiphilic and cationic characteristics that can cause membrane damage, leading to impaired function of surface and intramembrane proteins, disruption of lipid bilayer integrity, and alterations in cellular shape. Additionally, these medications have the potential to interfere with cellular energy generation processes. The latter is achieved through several mechanisms. For example, certain types of phenothiazines can disrupt the transportation of ions across the plasma membrane, leading to a decrease in membrane potential and proton motive force, which in turn affects the cell's ability to generate energy. This effect is not achieved by altering the permeability of the membrane, and it is associated with energy availability. Phenothiazines have been demonstrated to hinder the activity of ATPases as well and exhibit antibacterial properties on both eukaryotes and prokaryotes by causing disruption in the plasma membrane and affecting the function of membrane‐bound proteins [5, 6]. Eventually, it has been reported that antipsychotics may have the potential to influence and block cell wall synthesis via binding with penicillin‐binding protein 1A. Although this finding has been supported by a molecular docking assay (in silico), however, further investigation and experimental studies are needed [6]. Chlorpromazine, among the other antipsychotics, has been widely investigated in vitro. Moreover, these phenothiazine compounds have also been investigated in vivo studies on different species of either MDR or standard strains [6, 7, 8]. For instance, the antibiotic effect of chlorpromazine has been illustrated on methicillin‐resistant *Staphylococcus aureus* , *Acinetobacter baumannii* (ATCC 17978, A578, and A564), and *Mycobacterium tuberculosis* (H37Rv) [6, 7, 9, 10].

In the '90s, it was discovered that these medications additionally caused synergistic effects if employed with antibiotics. Since then, there has been more extensive research on the synergistic effect of antipsychotics on known antibiotics [11]. An illustration of the combined antibacterial impact of chlorpromazine on *Escherichia coli* has been proven. Research has discovered that chlorpromazine can enhance the vulnerability of *E. coli* (ATCC8739) to Brevinin‐2CE by exerting inhibitory effects on efflux pumps in resistant strains [9]. The combined impact of chlorpromazine and other conventional antibiotics like fluoroquinolone and colistin has been investigated recently [6]. Trifluoperazine and promethazine, which are additional derivatives of phenothiazine, have also been studied for their antibacterial properties, and the outcomes were found to be significant. Trifluoperazine has been studied for its potency against MDR *M. tuberculosis* (H37Rv, JAL2287, and 1934). It has shown antibacterial action either independently or when used with other antibiotics. The effect is achieved by exerting an antagonistic impact on calmodulin‐like protein and targeting the associated pathway [12].

Considering all the aspects described above, it is important to note that the focused use of drug repositioning targeting specific resistance mechanisms and bacteria would optimize the beneficial outcomes in overcoming various MDR infections. Therefore, antipsychotics are promising in the field of medication repositioning, given their broad range of mechanisms of action, as previously stated. While numerous studies have shown the beneficial application of antipsychotics in drug repositioning as antimicrobial medicines, several studies have specifically examined a certain antibacterial mechanism of action of these medications. For instance, promethazine is effective against *Burkholderia pseudomallei* via performing inhibitory impact on the efflux pumps, which is a known mechanism by which bacteria develop resistance. The administration of promethazine has been shown to impair biofilm synthesis and hence increase the susceptibility of this organism against antimicrobial agents [13]. Also, the plasmid‐encoded resistance gene is considered a possible mechanism for the development of bacterial resistance through transformation. In a separate study, the drug promethazine has been shown to be effective against various strains in mixed cultures by exerting anti‐plasmid effects [14]. Nevertheless, these are experimental potentials and are still not clinically validated. In conclusion, it seems that antipsychotics can exert an antibacterial effect with more than one mechanism in a living organism. However, further investigation and more focused administration are needed to target a certain mechanism that is responsible for resistance.

The current article provides a broad review of the various pathways proposed for the antibacterial activity of antipsychotics that have been identified (Figure 1). While there is experimental evidence that strongly supports several of these mechanisms, such as the inhibitory effects on the efflux pumps, some of these mechanisms have not been thoroughly investigated. For instance, the impact of phenothiazine on the respiratory chain in *M. tuberculosis* and the inhibition of cell wall production by chlorpromazine have been investigated by laboratory experiments and in silico approaches, respectively [15]. However, there is currently a lack of scientific evidence to fully substantiate these findings. In addition, the investigations conducted to examine the antibacterial impact of antipsychotics mostly consist of in vitro and in vivo experiments. The human body is composed of an array of commensal microbiomes known as microbiota. The human gut microbiota has the ability to impact the metabolism and absorption of various drugs. Furthermore, any antimicrobial drugs that impact pathogenic and MDR strains have the potential to also harm the commensal and beneficial microbes residing within a living organism. Considering the significance of gut microbiota in regulating several physiological processes [16, 17], it is possible that antipsychotics can cause harmful changes in the human gut microbiota, a condition known as dysbiosis. This is a significant and worrisome challenge, as numerous studies in recent years have demonstrated a strong connection between dysbiosis and a variety of illnesses [18, 19]. Antipsychotics' impacts on gut microbiota are variable; among them, olanzapine, for instance, is associated with gut dysbiosis and metabolic dysfunction; however, lurasidone has been found to be safe in rats regarding this matter [20, 21]. This variability should be considered to find better candidates in future studies. Antimicrobial drugs exert a selective force on bacteria, resulting in the emergence of resistant strains. This has been investigated on repositioned drugs as well, including the antidepressant [22]. Besides, while antipsychotics are effective for psychotic disorders, their use in non‐psychotic populations is concerning due to the lack of proven efficacy and the risk of adverse effects, including metabolic effects, movement disorders, and sleep problems [23, 24]. Thus, careful monitoring for adverse effects is essential when prescribing antipsychotics, especially in non‐psychotic patients, where the risk–benefit ratio is less favorable [25]. Moreover, since several antibacterial effects of phenothiazines are achieved in higher doses than therapeutic ranges approved for humans, repositioning antipsychotics as antimicrobial medicines may have the potential to cause detrimental effects [5]. Regardless of the obstacles, this avenue is worth scientific attention. Therefore, the main steps toward repurposing antipsychotics might be as follows: Initially, there is computational evidence (i.e., molecular docking) that is not fully validated in vitro or in vivo. After all, it is necessary to conduct more in‐depth studies, particularly more pre‐clinical and clinical studies, focusing on previous findings, to address obstacles regarding finding the best therapeutic dose, safety, and adverse effects, overall efficacy, and, especially given the pharmacology of these drugs, the infection type and spectrum within the human body.

**FIGURE 1:**
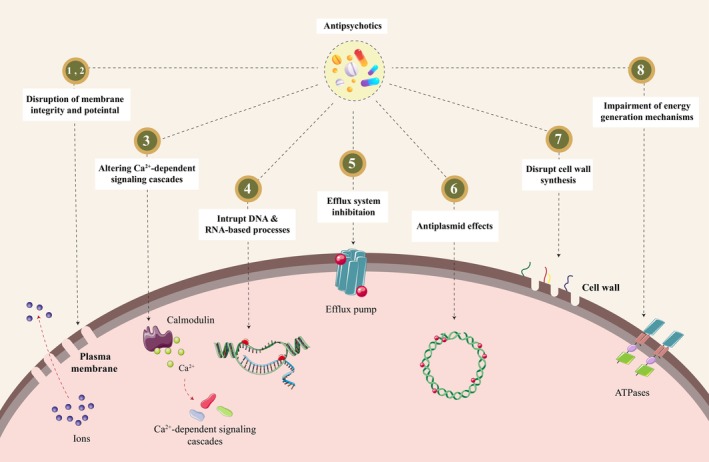
Antipsychotics can exert antibacterial effects via several mechanisms: (1, 2) Due to their amphiphilic and cationic characteristics, antipsychotics can negatively affect the integrity of the plasma membrane by disrupting its structure and electrochemical potential. Subsequently, cellular shape and intracellular ionic balance vanish leading to a decrease in bacterial viability. (3) Antipsychotics can interact with the protein calmodulin and change Ca^2+^‐dependent cascades. (4) Additionally, they possess the ability to hinder processes associated with nucleic acids, such as DNA replication and RNA translation. (5) Antipsychotics also target the efflux pump, a significant mechanism through which bacteria develop resistance. These medications can inhibit the efflux system by interacting directly with their subunits and altering their activity. (6) They may impede the replication of plasmids as well. (7) Additionally, they have been demonstrated to hinder cell wall formation by binding to PBP; however, more investigation is needed to clarify this mechanism. (8) Eventually, antipsychotics can exert their antibiotic effect by altering energy‐producing pathways via disruption in membrane‐bound ATPase pumps.

## Funding

The authors have nothing to report.

## Conflicts of Interest

The authors declare no conflicts of interest.

## Data Availability

The authors have nothing to report.
